# Human-to-Animal Transmission of SARS-CoV-2, South Korea, 2021

**DOI:** 10.3201/eid2905.221359

**Published:** 2023-05

**Authors:** Jinsun Bae, Changseek Ro, Yunhee Kang, Eulhae Ga, Woonsung Na, Daesub Song

**Affiliations:** Seoul Metropolitan Government, Seoul, South Korea (J. Bae, C. Ro, Y. Kang);; Chonnam National University, Gwangju, South Korea (E. Ga, W. Na);; Seoul National University, Seoul (D. Song)

**Keywords:** COVID-19, 2019 novel coronavirus disease, coronavirus disease, severe acute respiratory syndrome coronavirus 2, SARS-CoV-2, viruses, respiratory infections, zoonoses, reverse zoonosis, dog, cat, South Korea

## Abstract

To investigate SARS-CoV-2 transmission from humans to animals in Seoul, South Korea, we submitted samples from companion animals owned by persons with confirmed COVID-19. Real-time PCR indicated higher SARS-CoV-2 viral infection rates for dogs and cats than previously reported from the United States and Europe. Host-specific adaptations could introduce mutant SARS-CoV-2 to humans.

The risk for zoonoses (animal-to-human transmission) is increasing as human and wildlife habitats overlap with more human and animal migration and industrial food animals worldwide. Reverse zoonosis (human-to-animal transmission) also occurs (*1*–*5*), introducing the possibility that an animal could act as a carrier and reinfect a person.

According to South Korea government health policy, every confirmed human case of COVID-19 is reported to the regional public health center, and epidemiologic investigations began in February 2021. To determine possible human-to-animal transmission of SARS-CoV-2, we surveyed SARS-CoV-2 results for companion animals (dogs and cats) owned by persons with confirmed COVID-19 who were living in Seoul during February–November 2021. We assessed only companion animals for which owners were confirmed and for which owners had requested testing. A total of 375 companion animals (271 dogs and 104 cats) were tested for SARS-CoV-2 by real-time PCR.

When a companion animal exhibits suspected clinical signs and its owner requests a test, the Seoul city animal specimen collection team is dispatched to collect samples. For this study, the veterinarian managing the protection facility collected samples from companion animals whose owners had been confirmed to have COVID-19 and transferred the animals to separate protection facilities. Sampling was conducted according to guidelines of the World Organisation for Animal Health (*6*). Samples were collected by swabbing 3 locations on the animals: the oropharynx, nasal cavity, and rectum. The samples were transferred to individual virus transport media (1 mL), packaged in 3-layer biosafety packaging containers, and transported to the testing facility while refrigeration was maintained.

The COVID-19 diagnosis was established by using the real-time PCR method recommended by the World Health Organization to determine the presence or absence of SARS-CovV-2 virus antigens (*7*). Among the amplification genes, both RdRp (RNA-dependent RNA polymerase) and E genes were detected, indicating SARS-CoV-2 positivity; cycle threshold for each was <38. When PCR was positive for samples from >1 of the 3 sampling sites, the animal was determined to have a positive result.

Using SPSS Statistics 24 (IBM, https://www.ibm.com), we cross-tabulated and statistically analyzed the COVID-19 infection rate for companion dogs and cats owned by persons with confirmed cases of COVID-19. We found that 102 (27.2%) of 375 animals examined had positive results for SARS-CoV-2 infection: 65 (24%) dogs and 37 (35.6%) cats (Table). When we compared the positivity rates for the 2 species, we found that the positivity rate for cats was significantly higher than that for dogs (p<0.024).

**Table:** Positivity rates for companion animals owned by SARS-CoV-2–positive persons in study of human-to-animal transmission of SARS-CoV-2, South Korea, 2021*

Animal	Test results, no. (%)		χ^2^ distribution	p value
	Positive	Negative		
Dogs, n = 271	65 (24.0)	206 (76.0)	5.100	0.024
Cats, n = 104	37 (35.6)	67 (64.4)		
Total, n = 375	102 (27.2)	273 (72.8)		

*Positivity rate for cats was significantly (p = 0.024) higher than that for dogs.

We also investigated the rate of positivity detection according to sampling site. The positivity rate was higher for samples collected from the oropharynx (72.41%) and nasal cavity (84.85%) of dogs and from the oropharynx (83.33%) and nasal cavity (75.0%) of cats than from the rectum from either species (30.3% for dogs and 51.43% for cats).

This study reveals SARS-CoV-2 positivity rates of 24.0% for dogs and 35.6% for cats in South Korea, higher than rates previously reported from studies of dogs and cats. Although the animals in our study were already known to have been exposed to SARS-CoV-2 because their owners were confirmed to have COVID-19, the rate of positivity is high compared with rates determined in previous studies of animals with SARS-CoV-2–positive owners (*8*,*9*). This finding emphasizes the value and necessity of managing infectious diseases in companion animals as well as in humans because the risk for reverse zoonoses increases when companion animals are in prolonged and close contact with their owners.

Our study was limited by having been conducted with animals consigned to the protection facilities of the Seoul City Government and those whose tests were requested by their owners because of the animals’ clinical signs. Owner bias might have affected the population in this setting.

Our study could provide epidemiologically meaningful data for public health. As SARS-CoV-2 spreads as a pandemic, reverse zoonotic infections will continue, and viruses will mutate to adapt to the new host. For companion animals living near humans, continuous epidemiologic investigations and monitoring will be needed.
